# MicroRNA-138-5p regulates pancreatic cancer cell growth through targeting FOXC1

**DOI:** 10.1007/s13402-014-0200-x

**Published:** 2015-02-10

**Authors:** Chao Yu, Min Wang, Zhipeng Li, Jie Xiao, Feng Peng, Xingjun Guo, Yazhu Deng, Jianxin Jiang, Chengyi Sun

**Affiliations:** 1 Department of Hepatobiliary Surgery, Affiliated Hospital of Guiyang Medical College, Guiyang, Guizhou Province 550004 China; 2 Department of Biliary-Pancreatic Surgery, Affiliated Tongji Hospital, Tongji Medical College, Hazhong University of Science and Technology, Wuhan, Hubei Province 430074 China

**Keywords:** Pancreatic cancer, miR-138-5p, Cisplatin, FOXC1

## Abstract

**Purpose:**

The prognosis of pancreatic cancer ranks among the worst of all cancer types, which is primarily due to the fact that during the past decades little progress has been made in its diagnosis and treatment. Here, we set out to investigate the role of microRNA 138 (miR-138-5p) in the regulation of pancreatic cancer cell growth and to assess its role as putative therapeutic target.

**Methods:**

qRT-PCR was used to examine the expression of miR-138-5p in 8 pancreatic cancer cell lines and 18 primary human pancreatic cancer samples. A lentivirual vector containing miR-138-5p mimics (lv-miR-138-5p) was used to exogenously over-express miR-138-5p in the pancreatic cancer cells lines Capan-2 and PANC-1. The effect of this over-expression on cell proliferation was examined using an in vitro propidium iodide fluorescence assay. Capan-2 cells exogenously over-expressing miR-138-5p were transplanted into nude mice to examine its in vivo effect on tumor growth. A predicted target of miR-138-5p (FOXC1) was first validated using a luciferase assay and, subsequently, down-regulated by siRNA to assess its effect on pancreatic cancer cell growth.

**Results:**

We found that miR-138-5p was markedly down-regulated in both pancreatic cancer cell lines and primary human pancreatic cancer samples, compared to a human pancreas ductal epithelial (HPDE) cell line and normal pancreatic tissues, respectively (*P* < 0.05). In addition, we found that in the pancreatic cancer cells lines Capan-2 and PANC-1 lentiviral transfection of miR-138-5p mimicked up-regulation of the endogenous expression of miR-138-5p and, concomitantly, inhibited cancer cell proliferation (*P* < 0.05). The exogenous over-expression of miR-138-5p also led to a significant inhibition of tumor formation in vivo. Using a luciferase assay, we found that miR-138-5p directly targets FOXC1. In conformity with this notion, we found that FOXC1 was down-regulated upon miR-138-5p over-expression in pancreatic cancer cells. Finally, we found that silencing of FOXC1 by siRNA had an inhibitory effect on pancreatic cancer cell growth.

**Conclusions:**

Our data indicate that miR-138-5p may play an important role in regulating pancreatic cancer cell growth, possibly through targeting FOXC1. Over-expression of miR-138-5p may serve as a novel approach for the treatment of patients with pancreatic cancer.

## Introduction

Pancreatic cancer is the seventh leading cause of cancer death in China and the fourth leading cause of cancer death in the United States [1, 2]. In the United States alone more than a quarter million people die from pancreatic cancer each year. The prognosis of patients with pancreatic cancer is one of the worst of all cancer types, due to the fact that little progress has been made in its diagnosis and treatment during the past decades [3, 4]. Therefore, it is of major relevance to seek for novel methods to detect and treat patients with pancreatic cancer.

MicroRNAs (miRNAs) comprise a class of 19–23 nucleotides long non-coding RNAs that can regulate endogenous gene expression by inducing the degradation or translational inhibition of target mRNAs [5]. In recent years, miRNAs have been shown to be able to modulate various biological processes, including embryonic tissue development, neural differentiation and apoptosis [6–8]. Importantly, a growing body of evidence indicates that miRNA expression levels may directly be associated with cancer development [6, 9–14]. In pancreatic cancer patients, several miRNAs have been identified that are differentially expressed in tumor tissues compared to adjacent normal tissues [15]. Among them, microRNA-21 has been found to be up-regulated in primary pancreatic cancers [16] and to modulate pancreatic cancer cell apoptosis [17]. This miRNA may serve as a biomarker for patients with pancreatic cancer [18]. Another example is microRNA-34 that has been found to play an important role in regulating the growth of pancreatic cancer stem cells [19].

Up- or down-regulation of miR-138 has been shown to play an important role in regulating the growth and/or apoptosis of various cancers, including lung cancer [20], hepatocellular carcinoma [21] and leukemia [22]. A recent study has additionally shown that in the human pancreatic cancer cell line PANC-1 up-regulation of miR-138 may lead to a decrease in focal adhesion kinase (FAK) expression [23]. As yet, however, it remains to be established whether miR-138 has a direct role to play in pancreatic cancer.

Forkhead box C1 (FOXC1) is a member of the family of forkhead box (FOX) transcription factors, originally identified as being involved in Axenfeld-Rieger syndrome [24, 25]. In addition to its role in embryonic development, FOXC1 has also been shown to serve as a biomarker for breast cancer [26, 27]. Other studies have shown that up-regulation of FOXC1 may increase cancer cell invasion and may imply a poor prognosis in cancer patients [28, 29]. In case of pancreatic cancer, a high expression level of FOXC1 has been found to be strongly associated with the occurrence of ductal adenocarcinomas [30], but its exact role in pancreatic cancer development is still largely unknown.

Here, we found that the expression levels of miR-138-5p, the most common human isoform of miR-138, are significantly down-regulated in both primary human pancreatic cancers and human pancreatic cancer-derived cell lines. In addition, we found that exogenous over-expression of miR-138-5p inhibits pancreatic cancer cell growth, both in vitro and in vivo, and that siRNA-mediated silencing of FOXC1, a direct target of miR-138-5p, similarly inhibits pancreatic cancer cell growth.

## Materials and methods

### Cell lines and cultures

The pancreatic cancer-derived cell lines AsPC-1, BxPc-3, Capan-1, Capan-2, CFPAC-1, PANC-1, MIA PaCa-2 and SW1990 were purchased from the American Type Culture Collection and cultured in CS-C medium supplemented with 10 % fetal bovine serum. The human pancreas ductal epithelial cell line HPDE was obtained from Dr. M.S. Tsao (Ontario Cancer Institute, Ontario, Canada) and maintained as previously described [31]. Primary human normal pancreatic epithelial cells were obtained from Cell System (Washington, USA) and cultured in CS-C medium containing 10 % fetal bovine serum (FBS) according to the manufacturer’s instructions.

### Primary tissue samples

Primary pancreatic tissue samples were obtained from 18 patients through a surgical protocol to resect a portion of the pancreas in the Department of Hepatobiliary Surgery, Affiliated Hospital of Guiyang Medical College, China, from January 2013 to March 2014. This cohort included 10 male and 8 female patients. The mean age was 58.5 years (range: 34–67 years). Tumor stages were determined using the ENETS [29] and AJCC-UICC [30] pathological classification systems. All eighteen patients exhibited a clear biopsy- or radiography-based evidence of stage IV pancreatic carcinoma. Informed consent forms were obtained from all patients. Autopsies were performed as soon as possible after death (<8 h). Samples of primary pancreatic carcinomas, as well as adjacent normal pancreatic tissues, were quickly removed and a part of each sample was embedded in OCT compound and stored at −80 °C. From the cancer tissues, only those with a clear diagnosis of ductal adenocarcinoma were included in this study. All procedures used were reviewed and approved by the Ethics Committees of the Affiliated Hospital of Guiyang Medical College and the Affiliated Tongji Hospital.

### Quantitative real-time reverse transcription-PCR (qRT-PCR)

Total RNA and miRNA fractions were isolated from tissues and cell lines using Trizol reagent according to manufacturer’s protocol (Invitrogen, USA). Total RNA concentrations were measured using a NanoDrop ND-1000 spectrophotometer (NanoDrop Technologies) at 260 and 280 nm (A260/280), and examined using an Agilent 2100 Bioanalyzer (Agilent Technologies). Quantitative real-time reverse transcription-PCR (qRT-PCR) assays were performed using a TaqMan miRNA Assay according to manufacturer’s protocol (Applied Biosystems). The amplification conditions were: 40 cycles of 15 s at 95 °C and 1 min at 60 °C. The expression levels of miR-138-5p and FOXC1 were normalized by the expression level of the housekeeping genes *U6* and *GAPDH*, respectively.

### Lentivirus production and transfection

Hsa-miR-138-5p oligonucleotide mimics and its non-specific control were synthesized by Ribobio (RiboBio, Shanghai, China). The respective coding sequences were then amplified and cloned into pCDH-CMV-MCS-EF1-coGFP constructs (System Biosciences, California, USA) to generate a miR-138-5p mimics oligonucleotide vector (lv-miR-138-5p) and its non-specific control vector (lv-control). Then, according to manufacturer’s instructions, the lentiviral expression constructs and a pPACK packaging plasmid mix were co-transfected into HEK-293 T cells, viral lv-miR-138-5p and non-specific lv-control particles were collected and titers were determined. In brief, the protocol for lentiviral production was: (i) Day 1, for each plasmid to be transfected 7 × 10^5^ HEK-293 T cells were seeded in 5 mL medium in a 10 cm tissue culture dish at 37 °C, 5 % CO_2_ overnight, (ii) Day 2, the transfection cocktails were added to the cells for 12–15 h, (iii) Day 3, the culture media were replaced by fresh media to remove the transfection cocktails, (iv) Day 4, the culture supernatants, containing lentiviral particles, were collected by centrifugation, transferred to polypropylene storage tubes and stored at 4 °C . The lentiviral titers were determined using a Lenti-X qPCRtitration kit according to the manufacturer’s instructions (Clonetech, Mountain View, CA, USA). Finally, pancreatic cells were transfected with the lentiviruses using Lipofectamine 2000 (5 μl/ml) according to manufacturer’s protocol (Invitrogen, Foster City, CA).

### Cell proliferation and colony forming assays

Pancreatic cancer cells were transfected with either lv-miR-138-5p or lv-control lentivirus and seeded in 6-well plates for 24 h. Viable cells were then transferred at a density of 2 × 10^4^ cells per well to 24-well plates and cultured for another 72 h. Cell proliferation was evaluated by measuring the fluorescence intensity of propidium iodide (PI) as described previously [32]. At 24, 48, 72 and 96 h the fluorescence intensities were measured using a CytoFluor II multiwell plate reader (PerSeptive Biosystems). For the colony formation assay, cells were seeded in 6-well plates at a density of 1,000 cells per well and cultured in an incubator. The cultures were terminated when the clones were visible (~48 h), fixed in methanol, and stained with hematoxylin for counting under a light microscope. All experiments were performed in triplicate.

### In vivo tumor formation

Capan-2 cells were transfected with lv-miR-138-5p mimic or lv-control vectors for 24 h. Next, the cells were collected and subcutaneously inoculated into female athymic NCr-nu/nu nude mice (6-week old) using a 0.3 ml suspension containing 2 × 10^6^ cells. The resulting tumor volumes were calculated using the formula (length × width^2^)/2. All animal experiments were performed according to the protocol approved by the Animal Committees of the Affiliated Hospital of Guiyang Medical College and the Affiliated Tongji Hospital.

### Luciferase reporter assay

Routine PCR was performed on cDNA of Capan-2 cells to amplify the wild-type 3’-UTR and mutant 3’-UTR (modified miR-218 binding site, Fig. 5a) of FOXC1. Then, the wild-type and mutant 3’-UTRs were inserted into a luciferase reporter vector (pmiR-REPORT, Ambion, USA) to generate Luc-FOXC1 and Luc-FOXC1-mu constructs, respectively. These constructs were verified by DNA sequencing. The pmiR-REPORT control vector and the Luc-FOXC1 and Luc-FOXC1-mu vectors were co-transfected with β-galactosidase and Lv-miR-138-5p vectors into HEK-293 cells in 12-well plates using Lipofectamine 2000 according to the manufacturer’s protocol. Luciferase activities were examined after 24 h using a luciferase reporter assay system (Promega, USA) according to the manufacturer’s protocol. The signals were normalized to the β-galactosidase control vector activity. The experiments were performed at least in triplicate.

### Transfection of siRNA

A FOXC1-specific siRNA (si-FOXC1) and its non-specific scramble siRNA (si-NC) were purchased from Stanta Cruz (Santa Cruz Biotechnology, USA), and transfections were carried out with Lipofectamine 2000 according to manufacturer’s recommended protocol. Briefly, Capan-2 and PANC-1 cells were transfected with either si-FOXC1 (100 nM) or si-NC (100 nM) for 48 h, followed by examination of cell proliferation. The efficiency of the siRNA on knocking down FOXC1 expression was examined by qRT-PCR 48 h after transfection.

### Western blotting

After culture, cell lysates were collected using routine procedures and protein concentrations were determined using a BCA kit (Thermo Scientific). Then 20 μg protein was run on a 9 % SDS-PAGE gel at 70 V for 4 h, transferred to a nitrocellulose membrane and incubated with a primary anti-FOXC1 antibody (1:500, Santa Cruz Technology, Santa Cruz, CA). A horseradish peroxidase-ECL method was used for X-ray film exposure and measurement of FOXC1 protein expression levels. GAPDH was used as internal control.

### Immunostaining

Formalin-fixed paraffin-embedded sections (5 μM) were prepared from mouse xenografts. They were deparaffinized and incubated with primary antibodies directed against FOXC1 and human Ki67. The respective proteins were visualized using NexES automated stainers and the I-View Detection Chemistry system (Ventana Medical Systems, Tucson, AZ).

### Statistical analysis

In vivo tumor volumes were analyzed using one-way ANOVA. Other data were presented as the mean ± SD and evaluated with a Student’s *t*-test. Statistical significance was considered if *P* < 0.05 in any analysis. All experiments were performed at least three in triplicate.

## Results

### miR-138-5p is down-regulated in pancreatic cancer tissues and cell lines

MiR-138-5p, one of the mature isoforms of the miR-138 family, has previously been shown to play a critical role in many types of cancer [33–35]. Unlike miR-138-5p, the other known mature isoform of the miR-138 family, miR-138-1-3p, does not appear to exert any relevant biological functions in humans (based on search results of Google Scholar and PubMed). Therefore, we decided to focus in our study on miR-138-5p.

**Fig. 1:**
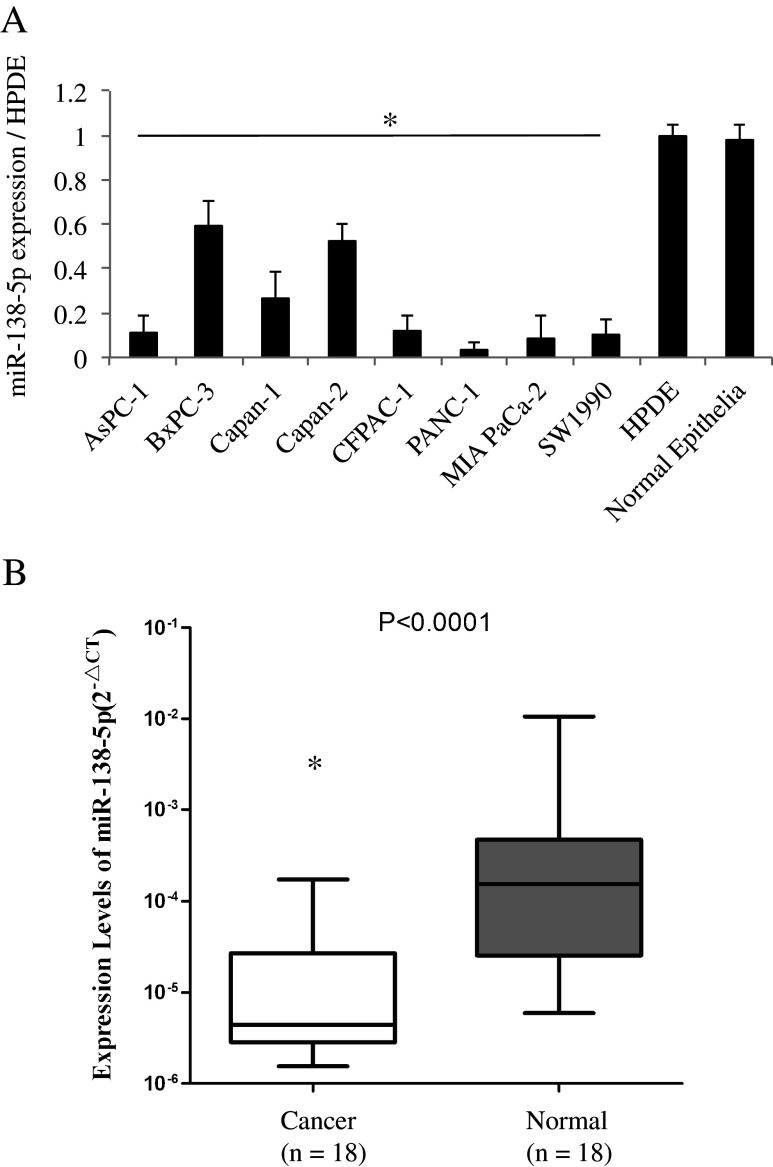
miR-138-5p expression levels in pancreatic cancer cell lines and clinical samples. **a** miR-138-5p expression levels in cell lines relative to HPDE and normal epithelia assessed by qRT-PCR (*, *P* < 0.05). Data are presented as Mean ± SD and the experiments were performed in triplicate. **b** miR-138-5p expression levels in 18 primary pancreatic cancer tissues relative to 18 nonmalignant (normal) tissues assessed by qRT-PCR (*, *P* < 0.05). Data are presented as Mean ± SD and the experiments were performed in triplicate

First, we used qRT-PCR to assess the expression level of miR-138-5p in 9 pancreatic cancer cell lines, a human pancreas ductal epithelial cell line (HPDE) and a primary culture of normal pancreatic epithelial cells (Fig. 1a). By doing so, we found that in all the pancreatic cancer cell lines tested the expression level of miR-138-5p was significantly lower than in the HPDE or primary normal pancreatic epithelial cells (*P* < 0.05). Next, we used qRT-PCR to assess the expression level of miR-138-5p in 18 primary pancreatic cancer tissues and 18 matched normal pancreatic tissues (Fig. 1b). Again, we found that the expression level of miR-138-5p in the pancreatic cancer tissues was significantly lower than that in the adjacent non-cancer tissues (*P* < 0.05).

### Over-expression of miR-138-5p inhibits pancreatic cancer cell proliferation in vitro

**Fig. 2:**
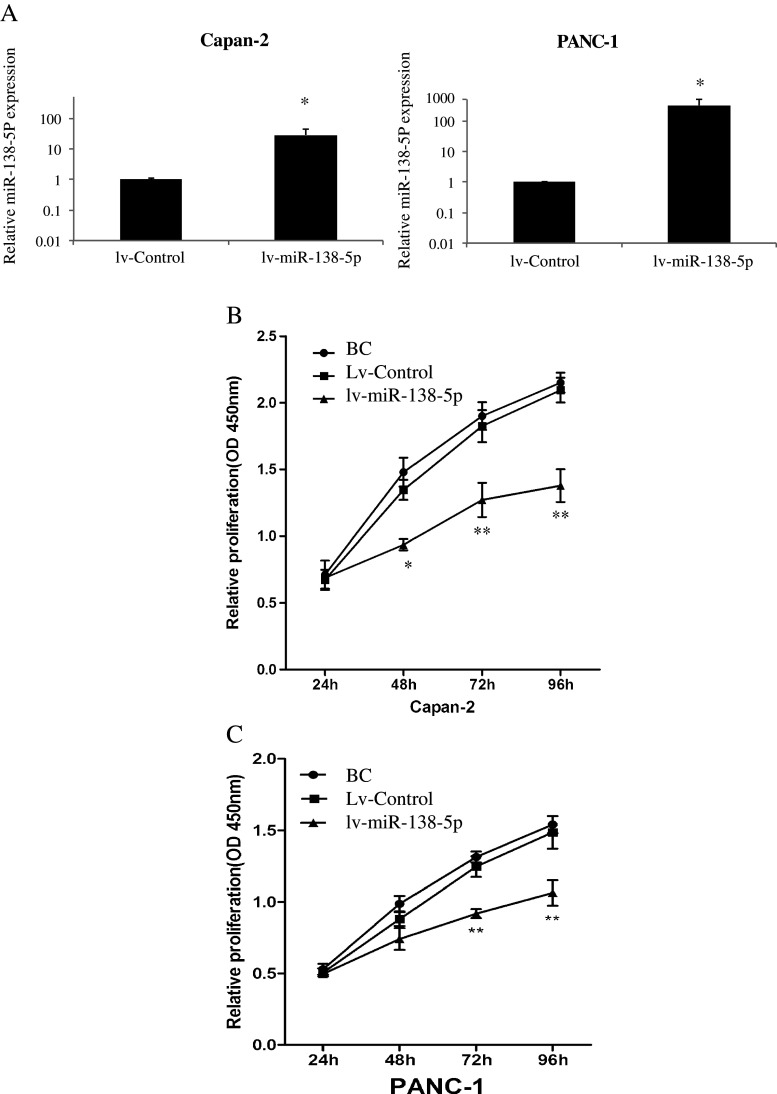
miR-138-5p inhibits proliferation of pancreatic cancer cells. Capan-2 and PANC-1 cells were transfected with a lentiviral vector expressing miR-138-5p mimics (lv-miR-138-5p, 100 pmol) and its non-specific control vector (lv-control, 100 pmol) for 48 h. **a** Endogenous expression levels of miR-138-5p after lentivirual transfection measured by qRT-PCR (*: *P* < 0.05). **b**, **c** Effects of miR-138-5p on cell proliferation of pancreatic cancer cells assessed by a cell proliferation assay at 24, 48, 72 and 96 h after seeding. Pancreatic cancer cells without lentiviral transfections were included (blank control, BC). In both Capan-2 (**b**) and PANC-1 (**c**) cells proliferation was significantly inhibited by lv-miR-138-5p at 72 and 96 h compared to lv-control (*: *P* < 0.05)

To better understand the functional role of miR-138-5p in pancreatic cancer, we used a lentivirual miR-138-5p mimics vector (lv-miR-138-5p) to exogenously up-regulate miR-138-5p expression. For this, two pancreatic cancer cell lines were used, Capan-2 and PANC-1. First, we verified the respective transfection efficiencies. The results obtained showed that miR-138-5p was significantly up-regulated in both transfected cell lines (Fig. 2a, *P* < 0.05). Next, we performed cell proliferation assays in both Capan-2 and PANC-1 cells. The results showed that, after transfection with lv-miR-138-5p lentivirus, the proliferation was significantly inhibited in both pancreatic cancer cell lines 48 h to 96 h after transfection (Fig. 2b, *P* < 0.05).

### Over-expression of miR-138-5p inhibits tumor formation in vivo

**Fig. 3:**
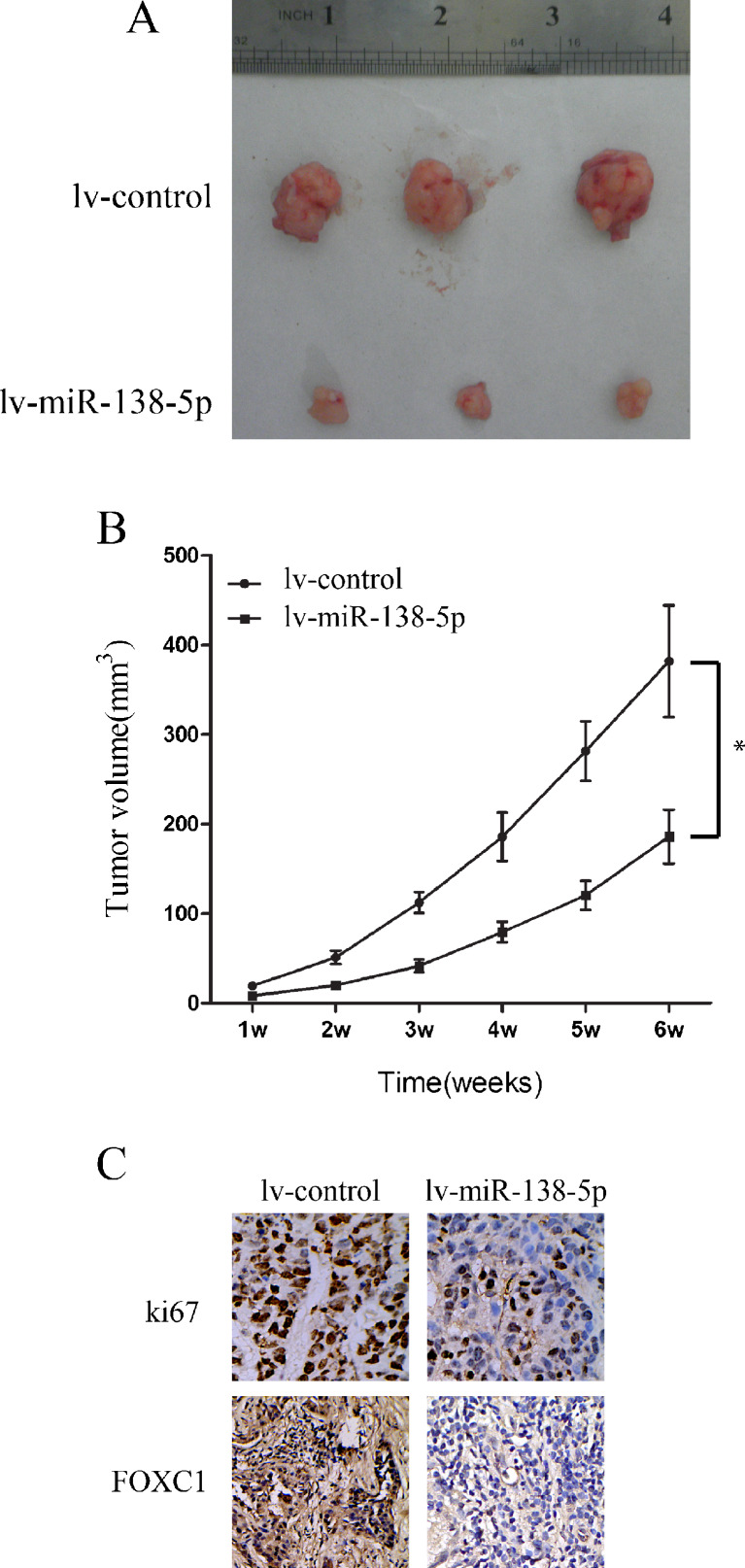
miR-138-5p over-expression inhibits Capan-2 tumor formation in nude mice. Capan-2 cells were transfected with lv-miR-138-5p or lv-control vectors for 24 h. Cells were collected and inoculated into female athymic nude mice. **a** Images of the tumors 6 weeks after inoculation. **b** Tumor volumes calculated using the formula: (length × width^2^)/2 from week 1 to week 6 (*: *P* < 0.05, one-way ANOVA). **c** Representative tissue sections after immunostaining for Ki67 and FOXC1

We then set out to examine the effect of exogenous miR-138-5p over-expression on pancreatic cancer growth in vivo. To this end, Capan-2 cells were transfected with lv-miR-138-5p or lv-control for 24 h. Next, the cells were collected and subcutaneously inoculated into female athymic nude mice. The results obtained showed that miR-138-5p over-expression significantly inhibited Capan-2 tumor growth in vivo (Fig. 3a-b, *P* < 0.05). Subsequent immunostaining for Ki-67 on tissue sections of mouse xenografts confirmed the inhibitory effect of miR-138-5p over-expression on tumor cell proliferation (Fig. 3c, top panel).

### miR-138-5p modulates pancreatic cancer cells through FOXC1

**Fig. 4:**
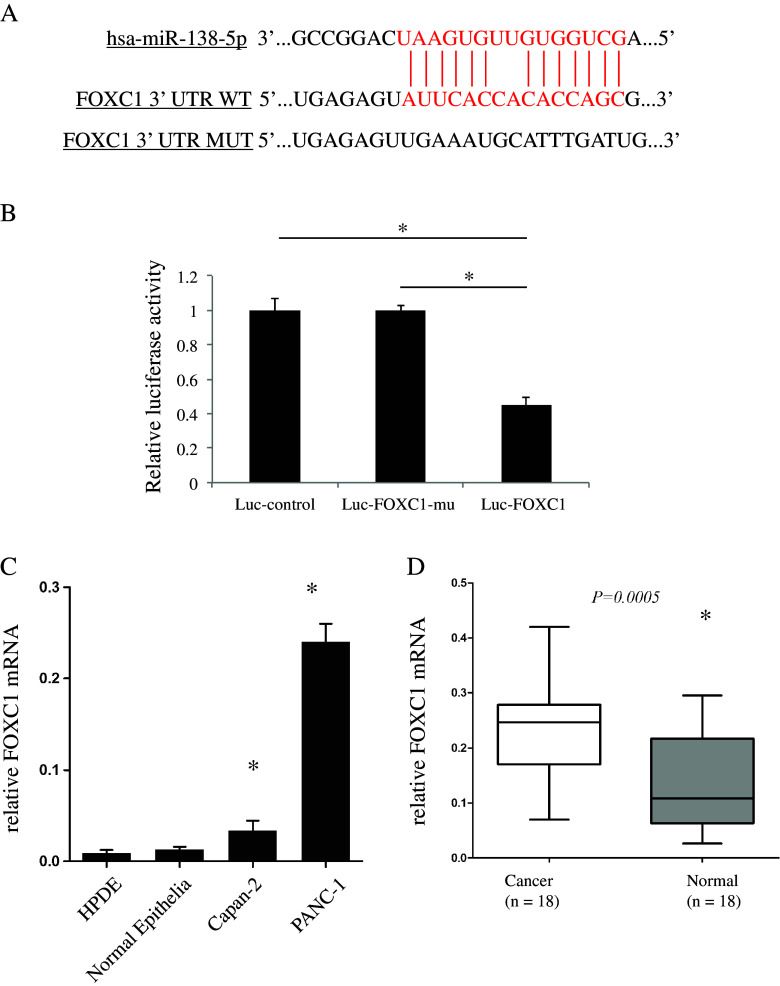
miR-138-5p directly targets FOXC1. **a** Predicted binding site of miR-138-5p in 3’-UTR of FOXC1. The mutated 3’-UTR of FOXC1 (FOXC1-mu) is also shown. **b** HEK 293 T cells were transfected with pmiR-REPORT control construct (Luc-control), mutant 3’-UTR FOXC1 construct (Luc-FOXC1-mu) or wild-type 3’-UTR FOXC1 construct (Luc-FOXC1), along with β-galactosidase and lv-miR-138-5p constructs. After 24 h, cells were examined by luciferase assay and the signals were normalized to β-galactosidase control vector activity in triplicates. (*: *P* < 0.05). After qRT-PCR, expression levels of FOXC1 were compared between normal pancreatic cells and pancreatic cancer cell lines **c**, as well as primary pancreatic cancers and normal clinical patient samples **d** (*: *P* < 0.05)

Finally, we set out to identify the molecular pathway (s) underlying miR-138-5p action in pancreatic cancer cells. Very recently, it was reported that FOXC1, a member of the family of forkhead box (FOX) transcription factors, was highly expressed in 85 patients with pancreatic ductal adenocarcinomas [30]. Through bioinformatic analyses, including TargetScan, Pictar and miRANDA, we found that FOXC1 may serve as a direct target of miR-138-5p (Fig. 4a). Even more interestingly, we found in the in vivo transplantation experiment that the expression of FOXC1 in the grafted miR-138-5p over-expressing tumor samples was significantly down-regulated (Fig. 3c, bottom panel). Based on these observations, we hypothesized that FOXC1 might be involved in the miR-138 mediated regulation of pancreatic cancer cell growth. To test this hypothesis, we used a luciferase assay and, by doing so, confirmed that miR-138-5p indeed directly targets FOXC1 (Fig. 4b). We also found by qRT-PCR that FOXC1 is up-regulated in Capan-2 and PANC-1 cells (Fig. 4c), as well as in primary pancreatic cancer tissues (Fig. 4d), compared to normal pancreatic cells (HPDE) and nonmalignant clinical tissues, respectively.

**Fig. 5:**
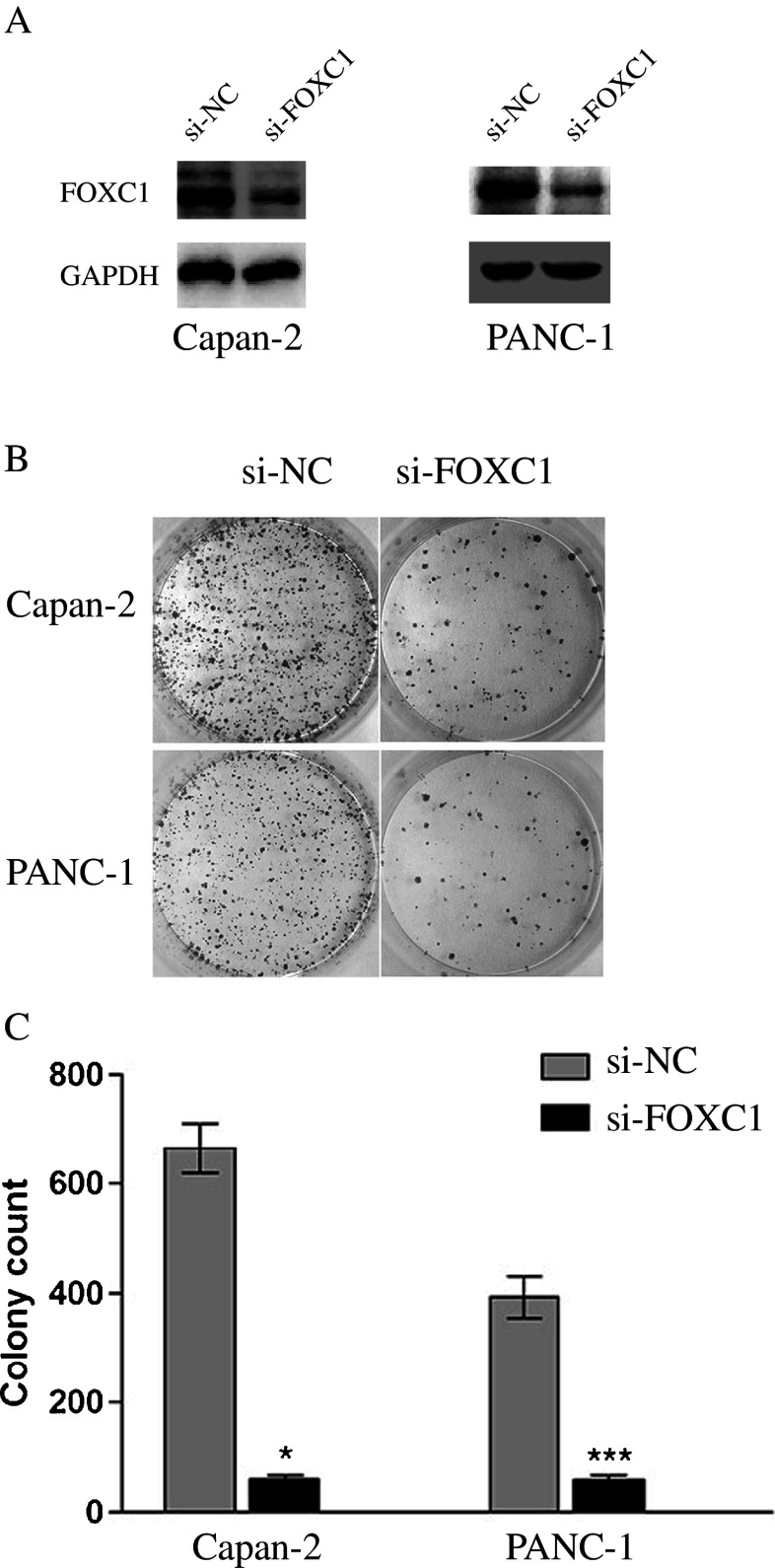
Similar effects of FOXC1 knock down and miR-138-5p over-expression on pancreatic cancer cells. **a** Capan-2 and PANC-1 cells were transfected with FOXC1 siRNA (si-FOXC1, 100 nM) or non-specific control siRNA (si-NC, 100 nM). The endogenous expression levels of FOXC1 were measured by qRT-PCR 24 h after transfection. **b** Colony formation assay performed on both Capan-2 and PANC-1 cells 48 h after transfection. **c** Colony numbers per dish compared between si-NC treatment and si-FOXC1 treatment (*, ***: *P* < 0.05)

We then applied siRNA to knock down FOXC1 expression in pancreatic cancer cells. To this end, 100 nM FOXC1 siRNA (si-FOXC1) or non-specific control siRNA (si-NC) were applied to Capan-2 and PANC-1 pancreatic cancer cell cultures for 24 h. The transfection efficiencies were checked by Western blotting and showed that in both cell lines the FOXC1 protein expression levels were significantly reduced after treatment with si-FOXC1 (Fig. 5a). We also found that the effects of knocking down FOXC1 were similar to up-regulating miR-138-5p, i.e., cell proliferation was significantly inhibited in both Capan-2 and PANC-1 as assessed in a colony-forming assay (Fig. 5b-c, *P* < 0.05).

## Discussion

Recently, large-scale genome, transcriptome and methylome analyses of pancreatic cancers was used to map the mutational landscape in more than 400 patients [36, 37]. In the present study, we show for the first time that the epigenetic modifier miR-138-5p is functionally involved in regulating pancreatic cancer cell growth. We show that miR-138-5p is generally under-expressed in pancreatic cancer cell lines and primary patient samples and, subsequently, that exogenous miR-138-5p over-expression can inhibit pancreatic cancer cell proliferation both in vitro and in vivo. These results are in line with previous studies showing that miR-138-5p can inhibit the growth of other cancer types and increase cancer cell chemo-sensitivity [20–22]. Our results also suggest that the molecular mechanism underlying miR-138-5p action in pancreatic cancer may be similar to its tumor suppressive role in other types of cancer, since miR-138-5p is generally down-regulated in pancreatic cancer tissues and since forced over-expression of miR-138-5p inhibits pancreatic cancer cell proliferation.

In the present study, we additionally show that FOXC1 may serve as a direct target of miR-138-5p in pancreatic cancer. We found that the expression levels of FOXC1 were significantly down-regulated by exogenous miR-138-5p over-expression both in vitro and in vivo, and that subsequent siRNA-mediated FOXC1 down-regulation yielded a similar effect in reducing pancreatic cancer cell proliferation as did over-expression of miR-138-5p. It has been shown by others that FOXC1 acts as a complex regulator in breast cancer, as both inhibiting and enhancing proliferative effects were observed after FOXC1 up-regulation [27, 38, 39]. In pancreatic cancer, it has been found that a high level of FOXC1 expression is strongly associated a with poor clinic outcome in patients with ductal adenocarcinomas [30]. Thus, our finding that FOXC1 is overall highly expressed in pancreatic cancer cell lines and primary pancreatic cancer tissues, as well our finding that FOXC1 down-regulation has an inhibitory (anti-proliferative) effect on pancreatic cancer cell growth, supports the idea that FOXC1 likely acts as a proliferative factor in pancreatic cancer.

In conclusion, we identified a new regulatory microRNA, miR-138-5p, in pancreatic cancer and uncovered a novel mechanism by which miR-138-5p acts to regulate pancreatic cancer cell proliferation. Up-regulation of the tumor suppressor miR-138-5p may serve as a new therapeutic option for patients with pancreatic cancer.
